# Navigating Precision Oncology: Insights from an Integrated Clinical Data and Biobank Repository Initiative across a Network Cancer Program

**DOI:** 10.3390/cancers16040760

**Published:** 2024-02-12

**Authors:** Bibek Aryal, Zhadyra Bizhanova, Edward A. Joseph, Yue Yin, Patrick L. Wagner, Emily Dalton, William A. LaFramboise, David L. Bartlett, Casey J. Allen

**Affiliations:** 1 Allegheny Singer Research Institute, Allegheny Health Network, Pittsburgh, PA 15212, USA; bibek.aryal@ahn.org (B.A.); bizhanova.jadira@med-kaznu.com (Z.B.); edward.joseph@ahn.org (E.A.J.); yue.yin@ahn.org (Y.Y.); 2 Division of Surgical Oncology, Institute of Surgery, Allegheny Health Network, Pittsburgh, PA 15212, USA; patrick.wagner@ahn.org; 3 Illumina, San Diego, CA 92122, USA; edalton@illumina.com; 4 Pathology, Allegheny General Hospital, Pittsburgh, PA 15212, USA; william.laframboise@ahn.org; 5 Allegheny Health Network Cancer Institute, Pittsburgh, PA 15212, USA; david.bartlett@ahn.org

**Keywords:** clinical data program, minimal common oncology data elements (mCODE), oncology biobank, personalized cancer care, genomic profiling

## Abstract

**Simple Summary:**

In order to advance cancer research and personalized care, the Allegheny Health Network Cancer Institute (AHNCI) established a clinical data program (CDP) consisting of a comprehensive biobank and data repository. This includes details on socio-demographic characteristics, diagnosis, tumor characteristics, treatments, and prognosis. By understanding individual patient characteristics, such as genetics, lifestyle, and environmental factors, researchers can determine more effective treatments and preventive interventions. The CDP aids in predicting therapy responses and clinical outcomes through the utilization of cancer-related biomarkers across various disease sites. The CDP supports the initiative by providing comprehensive patient information, such as demographic characteristics, diagnosis details, and treatment responses, which, when combined with genomic data, can enhance the understanding of disease progression and treatment outcomes, thereby facilitating personalized care and precision medicine.

**Abstract:**

Advancing cancer treatment relies on the rapid translation of new scientific discoveries to patient care. To facilitate this, an oncology biobank and data repository program, also referred to as the “Moonshot” program, was launched in 2021 within the Integrated Network Cancer Program of the Allegheny Health Network. A clinical data program (CDP) and biospecimen repository were established, and patient data and blood and tissue samples have been collected prospectively. To date, the study has accrued 2920 patients, predominantly female (61%) and Caucasian (90%), with a mean age of 64 ± 13 years. The most common cancer sites were the endometrium/uterus (12%), lung/bronchus (12%), breast (11%), and colon/rectum (11%). Of patients diagnosed with cancer, 34% were diagnosed at stage I, 25% at stage II, 26% at stage III, and 15% at stage IV. The CDP is designed to support our initiative in advancing personalized cancer research by providing a comprehensive array of patient data, encompassing demographic characteristics, diagnostic details, and treatment responses. The “Moonshot” initiative aims to predict therapy responses and clinical outcomes through cancer-related biomarkers. The CDP facilitates this initiative by fostering data sharing, enabling comparative analyses, and informing the development of novel diagnostic and therapeutic methods.

## 1. Introduction

In 2019, cancer was a leading cause of death in the United States, with the most prevalent cancer types being breast, prostate, lung and bronchus, and colorectal cancers [1]. In recent years, there have been significant strides in improving cancer care and advancing research. Between 1999 and 2019, there was a noticeable decline in the rates of these major cancer-related deaths [2]. However, the incidence of various cancer types has increased among individuals under 50 years old, which may be attributed to lifestyle changes, environmental hazards, and heightened genetic susceptibility [3]. This trend highlights the pressing need for a more tailored approach to personalized cancer treatments that consider individual characteristics, such as genetics, lifestyle, and environmental factors. 

Genomic profiling is crucial for personalizing cancer treatments and ultimately improving patient outcomes. For tumors, next-generation sequencing (NGS) is used to perform comprehensive genomic profiling (CGP), where hundreds of genes are assessed, including relevant cancer biomarkers and genomic signatures as established in guidelines and clinical trials, for therapy guidance [4]. NGS can also be used to identify DNA variations in patients with established familial cancer histories to determine those individuals with elevated cancer risk due to an inherited mutation [5]. Routine germline and somatic testing for appropriate patients enables the practice and benefit of precision medicine by identifying those patients who may be eligible for targeted therapy for their cancer, and high-risk screening and management options for those with hereditary risks [6]. Evidence from diverse studies calls for broader germline testing beyond familial cases [7,8,9,10,11,12]. Restricting germline genetic testing to a subset of cancer patients at high risk of developing hereditary cancer hampers clinical trial participation, worsens treatment disparities, restricts therapy, and prevents access for patients and at-risk families [11]. Bridging this gap in detecting relevant mutations requires a systematic collection of biological, genetic, and clinical data.

Sharing patient data from cancer care centers will better our understanding of individual patients’ needs and advance clinical practices that will improve patient outcomes [13]. However, significant hurdles impede the effective sharing of uniform clinical oncology information across care providers. Research-grade data are typically confined to the limited pool of patients engaged in clinical trials, necessitating labor-intensive and financially unsustainable manual data extraction from unstructured sources. This disparity is particularly pronounced in handling genomic data, as most electronic health records (EHRs) inadequately accommodate the demands of precision oncology.

The “Moonshot” program commenced in October of 2021 and is a collaborative effort spanning across all 21 Allegheny Health Network (AHN) Cancer Institutes, hospitals, and affiliated sites in Pennsylvania (Table 1). A pivotal component of this initiative is the establishment of the clinical data program (CDP) which was strategically devised to address existing disparities in accessing research-grade data. The CDP assumes a pivotal role within this initiative, systematically undertaking the collection, analysis, and dissemination of patient clinical, biological, and genetic data.

To ensure the uniformity and efficient exchange of collected data, we leveraged the Minimal Common Oncology Data Elements (mCODE) framework. This framework was collaboratively developed by a diverse group of experts under the guidance of the American Society of Clinical Oncology (ASCO), a federally funded research and development center (MITRE, Bedford, MA), a National Cancer Institute-sponsored clinical trials research consortium (Alliance for Clinical Trials in Oncology), and the US Food and Drug Administration. Operating as a consensus data standard for oncology, mCODE specifies a computable set of data elements based on clinical use cases (codex.hl7.org, accessed on 1 December 2023). It also functions as both a common language and a model, facilitating a comprehensive approach to patient care and informing research by enabling the analysis of data across the entire journey of a cancer patient and among diverse patient cohorts [14].

**Figure 1:**
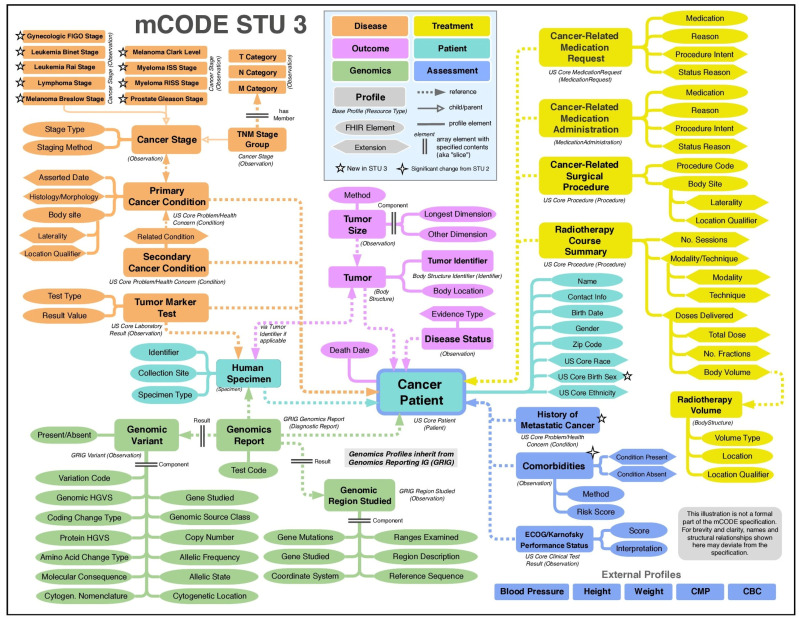
mCODE conceptual framework © 2024+ HL7 International. Reprinted with permission from HL7 CodeX under the Creative Commons license (source: codex.hl7.org, accessed on 1 December 2023).

Addressing the critical need for improved health data interoperability in oncology, mCODE serves as an open-source set of structured data elements, establishing minimum standards for health record information. The framework enables integration of clinical, biological, and genetic data (Figure 1), fostering a holistic approach to personalized cancer care. Leveraging the Fast Healthcare Interoperability Resources standard, mCODE ensures standardized and efficient information exchange. The ability to share patient data by engaging diverse stakeholders and harnessing existing data standards benefits both clinical care delivery and cancer research [15].

To provide a broader context for our biobanking initiative, we acknowledge the noteworthy achievements of established large-scale biobank integration efforts. The European Cancer Moonshot Center [16] and the UK Biobank stand as significant endeavors in the field [17], contributing valuable insights to the landscape of biobanking in oncology research.

In this report, we provide a comprehensive overview of the oncology biobank and data repository program, with a specific emphasis on the CDP within this initiative. The primary objective is to highlight the development of the CDP. We share the methods, challenges, and impact the CDP has on advancing cancer research and improving personalized care. 

## 2. Materials and Methods

### 2.1. Building the Oncology Biobank and Data Repository

AHN patients identified by their physician as either being likely to have or having cancer are eligible for enrollment. Identification occurs during routine medical care at all AHN Cancer Institute offices and affiliate offices by the physician or the oncology care team. Potential participants are approached with detailed information about the trial and the biobank during their medical consultations. This process ensures that individuals have a comprehensive understanding of the study, including the purpose, potential risks, and benefits. Written informed consent is obtained from the participants, granting AHN permission to collect longitudinal data up to 16 years later. We have recorded screening and recruitment numbers from 2020 to 2023. Patient samples and data are stored within laboratory facilities situated across AHN and its partner sites. Study enrollment will conclude upon reaching an approximate cohort size of 10,000 subjects.

Patients who have agreed to voluntarily provide a blood sample are then asked to contribute approximately 40–50 mL of additional blood for the biobank. This blood draw preferably occurs when patients are already having blood drawn at an AHN or AHN-affiliated draw site for standard-of-care labs. For surgical patients, a 50 mL (whole) blood draw is performed around the time of surgery, while for other patients, a 40 mL blood draw is scheduled at appropriate times under the direct supervision of the PI and/or delegated key study personnel. 

**Figure 2:**
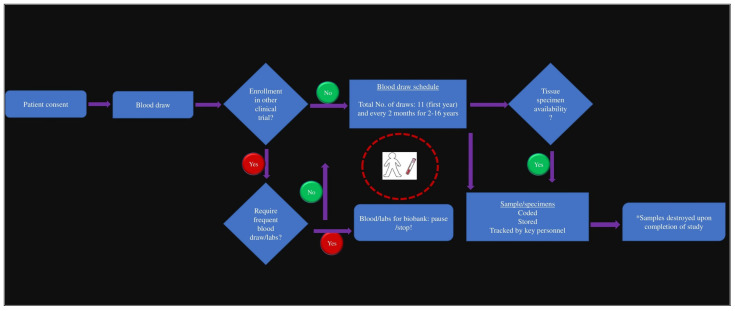
Illustration of patients’ enrollment, sample acquisition protocol, and biobank processing. * according to Institutional policy.

Additionally, a subset of consenting patients, specifically those undergoing surgical tumor resection, is asked to provide portions of the tumor and neighboring lymph nodes, if available, for further analysis. The collection of these biomaterials follows a specific protocol to ensure both quality and relevance to the research. For those unable to provide immediate samples, archival tissues are used. All specimens undergo coding for secure storage, with exclusive access granted to authorized personnel. This comprehensive protocol ensures secure, ethical, and patient-centric collection, storage, and utilization of tissue specimens for advancing oncology research (Figure 2).

### 2.2. Developing the Clinical Data 

**Figure 3:**
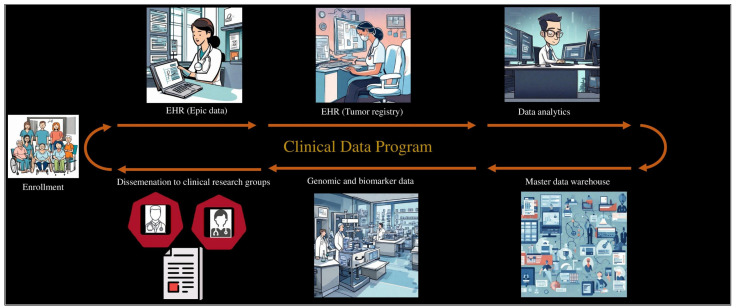
Clinical data workflow: Patient enrollment initiates data extraction from Epic/electronic health record (EHR) and tumor registry. Following analytics, a master dataset is formed and forwarded to the genomic and biomarker lab. Processed data are dispatched to the clinical research teams.

The development of clinical data involves a process orchestrated by a dedicated team of data stakeholders. This team extracts and integrates comprehensive patient information from 6 key data sources: Epic Clarity, EPIC reports, genomic lab data, biomarker data, and the AHN Oncology Registry. Epic Clarity incorporates enrollment details, consent dates, and comorbidities, while EPIC captures and reports crucial temporal aspects related to blood sample dates. Genomic lab data delve into genetic nuances, and the biomarker data group contributes insights into early detection, risk stratification, prognostication, and thereby informing personalized and timely interventions. The Oncology Registry provides essential cohort- and cancer-related information. Figure 3 illustrates the framework of development and dispatch of clinical data.

We use the mCODE framework to ensure the uniform and efficient exchange of the collected data [15]. Implementing the mCODE framework in the CDP is a strategic decision to ensure the quality of oncology data. It ensures that the data align with recognized interoperability standards which enhance meaningful utilization across diverse platforms and institutions. A bi-weekly meeting is conducted with the dedicated team of data stakeholders to ensure streamlined data integration, creating a central master dataset aligning with the mCODE framework. This adaptive architecture promotes continuous improvement and addresses challenges in data engineering and governance. 

The confidentiality of all data and records generated throughout this study will be maintained in accordance with institutional policies and HIPAA guidelines on subject privacy. The utilization of such data and records for purposes other than conducting the study or collaborating with fellow researchers will be strictly prohibited by the investigator and other site personnel.

## 3. Results

**Figure 4:**
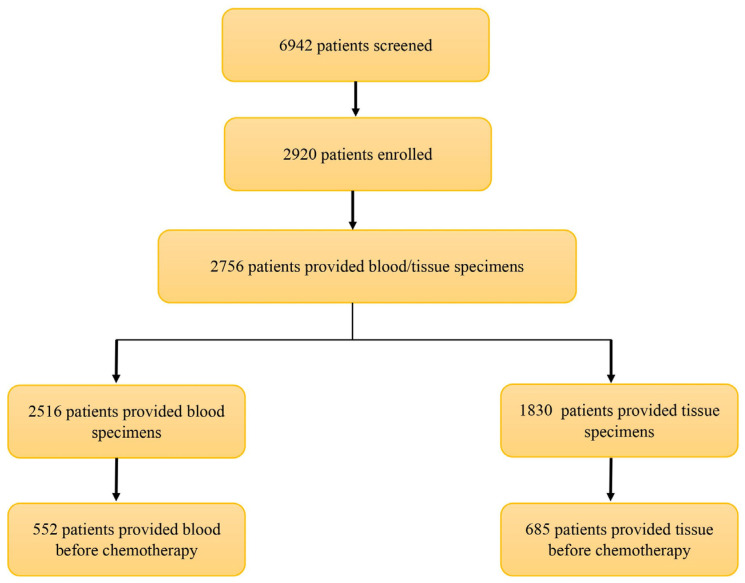
The flow chart illustrates patient enrollment status in the Moonshot program in the Allegheny Health Network. Of the 6942 patients screened so far, 2756 of them provided blood and/or tissue specimens.

**Figure 5:**
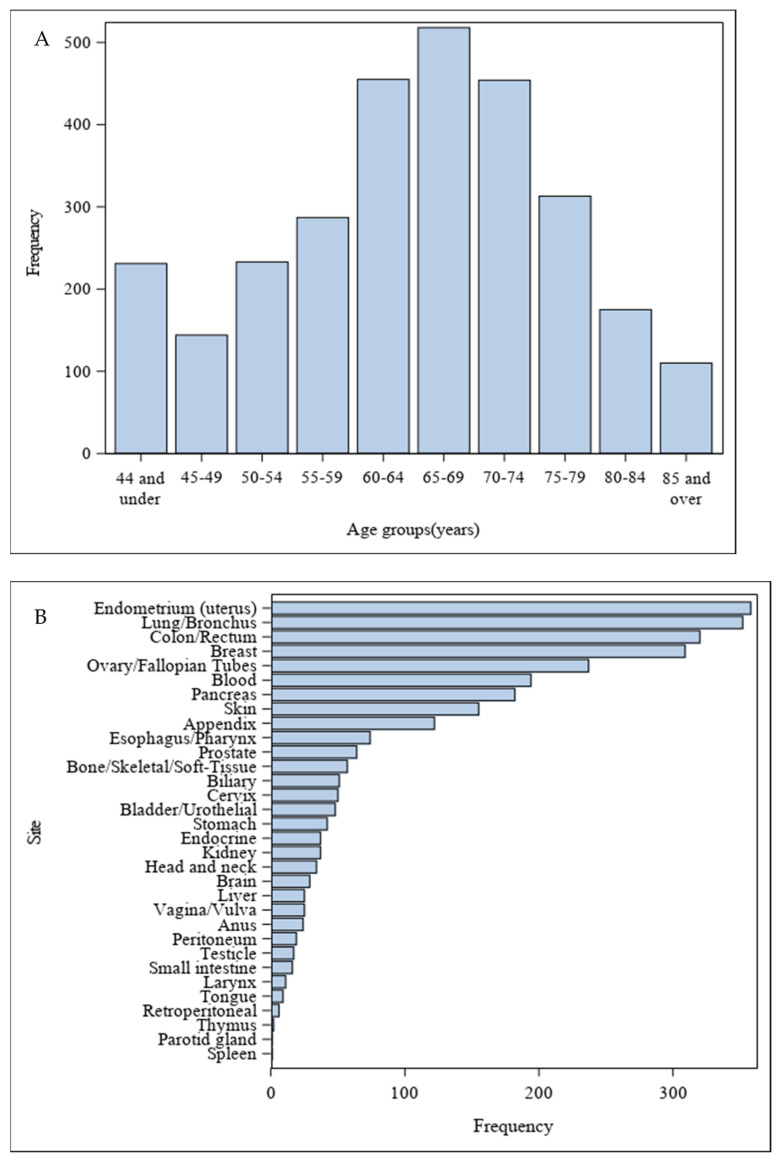

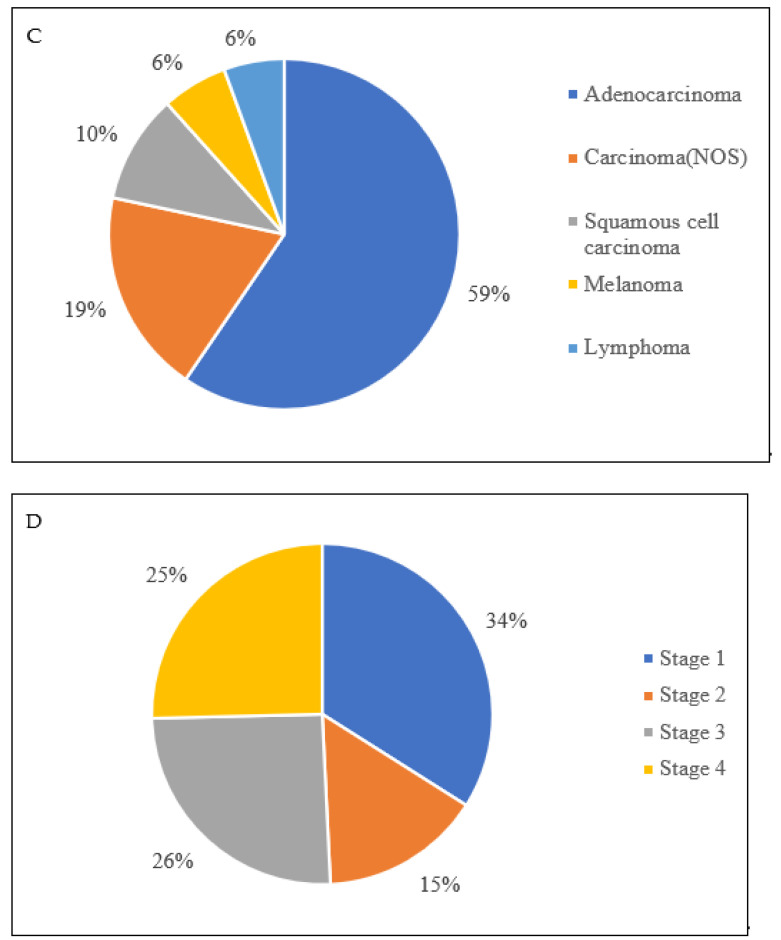
Demographics and clinicopathological data of patients enrolled in the Moonshot program: (**A**) Distribution of patients by age groups. (**B**) Distribution of cancer sites. (**C**) Distribution of common tumor histologies. (**D**) Distribution of tumor stages.

Our program has enrolled 2920 patients out of 6942 screened individuals. Among the 2756 patients who provided blood or tissue specimens, 552 contributed blood samples (blood group) and 685 provided tissue specimens (tissue group) prior to treatment (Figure 4). Figure 5 demonstrates the general demographics and clinicopathological characteristics. The study participants had a mean age of 64 ± 13 years. The majority of participants were female (61%) and Caucasian (91%). In the total population, the most common cancer sites were the endometrium/uterus (12%), lung/bronchus (12%), breast (11%), and colon/rectum (11%). The common primary cancer histology types included adenocarcinoma (60%), carcinoma not otherwise specified (NOS) (19%), squamous cell carcinoma (10%), and melanoma (6%). Across the stages, 34% were diagnosed at stage I, 25% at stage II, 26% at stage III, and 15% at stage IV.

For patients who provided both blood and tissue specimens to the Genomics wing of the program, NGS and ctDNA testing revealed a high level of agreement (97.0 ± 0.9%) in detecting genomic variants in both tumor tissue and correlative blood samples. Additionally, ctDNA assays identified specific mutations that were undetected in tumor specimens likely due to tumor heterogeneity, highlighting the potential for blood-based biomarker discoveries pertinent to patient care [18].

## 4. Discussion 

Establishing an ideal dataset for longitudinal studies of cancer treatment and outcomes is a complex challenge, and no clear consensus has yet emerged on what would constitute the optimal set of clinical and underlying biological information. Existing population datasets, such as the National Cancer Database (NCDB), are fundamentally important in epidemiologic research into broader trends in cancer incidence and treatment. However, they have serious limitations in the integration of tumor biology and treatment response at the level of individual patients. The existing national databases lack longitudinal treatment data, critical for a comprehensive understanding of treatment outcomes. Furthermore, the dearth of clinically relevant endpoints within the database hinders specific types of research and analysis. Challenges related to interoperability and data standardization further complicate data collection across diverse institutions. Significantly, the unique constraints of the existing big databases preclude the extraction of conclusive findings from a singular source [19]. This vacuum has led many cancer centers to devise idiosyncratic “homegrown” datasets to support their research efforts in clinical oncology. The CDP has addressed this gap by integrating clinical and biological data into a comprehensive format, updated in real time during routine clinical practice, for identifying and validating biomarkers. Longitudinal surveillance of disease facilitates evaluation of treatment, evaluation of cancer progression, and identification of signs of recurrence [20]. Moreover, this initiative promotes advances in genomics, proteomics, and other new technologies that enhance our understanding of the molecular properties of cancer. 

The CDP aims to address known issues with data collection. Data collected during routine medical care lack a research-ready structure: stained tissue specimens often require manual location and scanning, while radiological images are stored in systems with limited clinical annotation. Data modeling is also complicated by institution-specific biases ranging from technical variation to discrepancies in clinical data ontologies. In addition, data collection initiatives are often restricted by barriers to long-term data acquisition, including patient compliance and trust in the program, accessing granular clinical data from disparate healthcare systems, and logistically tracking and following patients over extended periods [21].

Another important consideration is a lack of ethnic diversity. Biobanks tend to exclude Indigenous people, socially disadvantaged individuals, and those with diverse cultural and linguistic backgrounds [22]. The predominant enrollment of Caucasian and female participants in our data collection highlights a discernible pattern in the demographic composition. It is crucial to acknowledge that such trends may not fully capture the diverse spectrum of cancer types and their prevalence among various ethnic groups, thereby emphasizing the necessity to explore and address potential disparities in cancer incidence and outcomes across different demographics. Addressing the ethnic gap in our biobank is vital for upholding ethical standards, enhancing the scientific validity of research, promoting health equity, and ensuring that the benefits of biobank research are accessible and applicable to all individuals. 

As our program expands through its affiliated network sites along different states, we are committed to implementing strategies to enhance diversity and inclusion. Collaborative efforts with healthcare systems and research institutions include tailored communication, education, and continuous evaluation aimed at addressing the under-representation of certain ethnic groups. The ongoing commitment to inclusivity is integral to the program’s evolution, ensuring that our research practices are not only diverse but also representative of the broader population. As we extend our reach, our goal is to establish a more comprehensive and inclusive dataset that accurately reflects the diversity of individuals affected by cancer across various demographic backgrounds, fostering a truly equitable foundation for advancing cancer research and personalized care.

### 4.1. Data Standardization

Data standardization is a core function of the CDP and will aid us in addressing the challenges listed above. The elements of mCODE are designed using standard, widely available medical terminology to enable searchability. This meticulous approach to data collection encompasses various facets of clinical information, socio-demographic characteristics, and diagnosis and treatments details. However, not all EHRs use mCODE, which limits its interoperability [10]. While the CDP utilizes the mCODE framework to harmonize clinical data, the mCODE project is still in its early stages with pilot implementations actively in progress. Our program not only underscores the novelty of employing mCODE in the context of longitudinal cancer studies but also contributes to the evolving landscape by actively implementing and refining this framework to bridge existing gaps in comprehensive data integration.

The CDP consolidates disparate sources and concurrently ensures the uniformity and compatibility of the information. This has allowed us to develop a standardized and interoperable data exchange process. However, the framework may not consistently accommodate all the variations in how cancer data are collected and stored across different institutions and systems. Therefore, integrating clinical cancer data in a multimodal context requires data engineering and curating and provisions for data access and governance. These challenges apply to both retrospective studies aiming to identify biomarkers from standard-of-care data and prospective studies concentrating on tailored data types.

By providing a master data repository, the CDP has not only streamlined internal operations but has also become a valuable resource for researchers by building a computable set of data elements based on clinical use cases. This can advance future cancer research and personalized cancer care, especially since the data repositories can be leveraged across time. By capturing data points at various time points, the CDP facilitates longitudinal data analysis, enabling a nuanced understanding of the dynamic aspects of cancer progression and treatment responses. The integration of multimodal data—ranging from genomic profiling to radiological and histological imaging—will offer a holistic understanding of cancer dynamics. This comprehensive analysis of every cancer is critical in achieving increased levels of precision in cancer treatment [23,24].

For instance, the advancement in genomic profiling of tumor tissue has significantly improved the precision of clinical decision-making, and the resulting genomic data serve as a valuable molecular repository for further investigations [25]. Subsequently, this fosters more comprehensive insights into the cancer genome, drug sensitivity [26], resistance mechanisms [27], and their prognostic implications [28]. Likewise, studies in cancer progression and recurrence are supplemented by the increasingly digitalized serial radiological images, tissue specimen profiling, and before and after intervention documentation [29].

### 4.2. Artificial Intelligence and Machine Learning

In the ever-evolving landscape of data-driven oncology, artificial intelligence (AI) has emerged as a transformative tool. Machine learning tools are essential for interpreting complex omics data, posing new challenges to the field and leading to a transformative change in liquid biopsy research [30]. Moreover, the dynamic and evolving nature of cancer treatment necessitates adaptive models that can continuously learn from new information. The fundamental prospect of multimodal data integration is that the data derived from different sources complement each other, thereby enhancing the information content beyond what any single source can provide [29].

The CDP operates in the realm of multimodal integration of clinical data, with advanced molecular diagnostics, radiological, and histological imaging, to advance precision oncology beyond certain genomic and molecular techniques. This integration in the CDP not only provides a holistic perspective on cancer dynamics but also facilitates the development of a new category of multimodal biomarkers driving innovations in the field of precision oncology. These complementary datasets provide an opportunity to learn from the collective history of large cohorts of patients, facilitating innovative personalized cancer care. As AI applications in clinical oncology continue to advance, the implications of AI and its use in digital pathology, biomarker development, and treatment optimization present both integration challenges and unprecedented opportunities [31].

### 4.3. Next Steps

Establishing collaborations between healthcare systems and research institutions is crucial for facilitating improved data sharing and access. These partnerships with other biobanks, data repositories, and research consortia worldwide will promote data sharing and collaborative research efforts and expand the program to a global initiative. Such work is already underway: Investigators in biomarker studies were supported by the master dataset provided by the CDP. Likewise, the CDP empowered a team working on a breast cancer project by disseminating comprehensive data on breast cancer patients.

We acknowledge the limitations of the current manuscript in terms of providing direct links to the study repository and detailed real-world examples illustrating the impact of the CDP. As the initiative is in its early stages, our primary focus has been on establishing the foundation for a comprehensive biobank and data repository. However, future studies will address these limitations by incorporating direct links to the study repository, providing more in-depth and interactive engagement for readers. Additionally, we are actively working on accumulating real-world examples and success stories to underscore the practical implications of the CDP on medical knowledge, patient outcomes, and scientific advancements. Our commitment extends to providing precise instructions for accessing and creating value, thereby enhancing the inherent user-friendly experience within our initiative.

## 5. Conclusions

In conclusion, the development of the CDP showcases a proof of concept for the effective integration of clinical, biological, and genetic data in a large integrated cancer network. As the program evolves, the continuous refinement of data collection methods and data sharing adopted by the CDP will propel the AHN “Moonshot” initiative towards its goal of advancing cancer research and personalized care. The venture set by the CDP is not just a scientific pursuit: it is a commitment to innovation and a beacon of hope for countless patients.

## Figures and Tables

**Table 1:** Allegheny Health Network hospitals and research sites.

Site Code	Site Name
AHNCI—AGH	AHN Cancer Institute—AGH
AHNCI—B	AHN Cancer Institute—Beaver
AHNCI—BU	AHN Cancer Institute—Butler
AHNCI—C	AHN Cancer Institute—Canonsburg
AHNCI—F	AHN Cancer Institute—Forbes
AHNCI—GC	AHN Cancer Institute—Grove City
AHNCI—H	AHN Cancer Institute—Hempfield
AHNCI—J	AHN Cancer Institute—Jefferson
AHNCI—NC	AHN Cancer Institute—New Castle
AHNCI—SV	AHN Cancer Institute—Saint Vincent
AVH	Allegheny Valley Hospital
BPH&WP	Bethel Park Health & Wellness Pavilion
CH	Canonsburg Hospital
FN	Federal North
Forbes	Forbes
GCH	Grove City Hospital
H&WP Erie	Allegheny Health & Wellness Pavilion Erie
JH	Jefferson Hospital
PTH&WP	Peters Township Health & Wellness Pavilion
SG	Suburban General
SVH	Saint Vincent Hospital
WAGH	WPAON—Allegheny General
WAV	WPAON—Allegheny Valley
WBO	WPAON—Butler Office
WF	WPAON—Forbes
WH&WP	Wexford Health & Wellness Pavilion
WHO	WPAON—Hansen Office
WJO	WPAON—Jefferson Office
WNC	WPAON—New Castle
WPH	West Penn Hospital
WPO	WPAON—Peters Office
WPU	WPAON—Punxsutawney
WR	WPAON—Robinson
WWP	WPAON—West Penn

AHN, Allegheny Health Network; AGH, Allegheny General Hospital; WPAON, West Penn Allegheny Oncology Network.

## Data Availability

Data not contained within the article are available on request.
